# Effect of Post-Exercise Whole Body Vibration with Stretching on Mood State, Fatigue, and Soreness in Collegiate Swimmers

**DOI:** 10.3390/sports5010007

**Published:** 2017-01-13

**Authors:** Justin J. Merrigan, Matthew N. Tynan, Jonathan M. Oliver, Andrew R. Jagim, Margaret T. Jones

**Affiliations:** 1 Health and Human Performance, George Mason University, Manassas, VA 20110, USA; jmerrig2@gmu.edu (J.J.M.); m.tynan@tcu.edu (M.N.T.); 2 Kinesiology Department, Texas Christian University, Fort Worth, TX 76129, USA; jonathan.oliver@tcu.edu; 3 Exercise and Sport Science, University of Wisconsin-La Crosse, La Crosse, WI 54601, USA; ajagim@uwlax.edu; 4 Center for Sports Performance, George Mason University, Fairfax, VA 22030, USA

**Keywords:** BAM, post-exercise recovery, ratings of perceived exertion, RPE

## Abstract

Static stretching (SS) during whole body vibration (WBV) has been suggested for exercise recovery. The purpose was to compare post-exercise self-ratings of fatigue (FAT), mood state (BAM), soreness (SOR), and perceived exertion (RPE) between SS and WBV+SS in swimmers (9 women, mean ± SD: 19.3 ± 1.3 year, 171 ± 5.7 cm, 67.6 ± 7.2 kg, 26.6 ± 4.1 %body fat (%BF); 10 men, mean ± SD: 19.7 ± 1.0 year, 183 ± 5.5 cm, 77.1 ± 4.2 kg, 13.1 ± 2.2 %BF). Athletes were divided by sex, event (sprint, distance), and assigned to SS or WBV+SS. Both conditions consisted of SS performed on the WBV platform with or without WBV (50 Hz, 6 mm). Sessions consisted of: pre and post measures of BAM, FAT, SOR; the condition; and RPE. Mixed factorial ANOVA were run. A significant condition by pre/post interaction was observed (*p* = 0.035). Post hoc analyses showed WBV+SS elicited lower post-exercise ratings of FAT (*p* = 0.002) and the BAM affective states, of tension (*p* = 0.031), and fatigue (*p* = 0.087). RPE did not differ between conditions. Of interest is the decrease in tension and fatigue noted by the BAM. Mood state can be indicative of how athletes adapt to training volume and intensity.

## 1. Introduction

Whole body vibration (WBV) is a mechanical stimulus, which is characterized by oscillatory motion, and is hypothesized to increase neuromuscular stimulation via increased muscle spindle activity and a lowered firing threshold [1,2,3]. WBV vibration platforms transfer energy to the human body through intervals of sinusoidal vibrations [2,4]. The intensity of WBV is determined by frequency (rate of oscillation cycles, Hz) and amplitude (displacement or extent of oscillatory motion, mm) [2,4]. The efficacy of WBV upon muscular strength, power, and flexibility is equivocal, and the optimal protocol for use has not been elucidated [1,2,3,4,5,6,7,8]. Reasons for the lack of clarity may be differences reported in the research in regard to experimental designs, vibration platforms, vibration parameters, fitness levels of participants, and individual neuromuscular and physiological responses [4,5,7]. Although popular with practitioners as a post-exercise recovery modality, the effects of WBV on flexibility, exercise-induced soreness, and fatigue have not been studied extensively.

Static stretching has been shown to reduce soreness and increase flexibility when performed pre- or post-exercise [9,10]. During static stretching, the stretches are often held slightly beneath the pain barrier, or when tightness is felt without pain, subsequently resulting in improved muscle elasticity [7,11]. The use of WBV has been shown to improve flexibility when used as a recovery aid [6,12]. Further, consistent static stretching performed in conjunction with WBV has also been shown to improve flexibility [6,7,8], albeit through different mechanisms. The increases in flexibility observed with WBV are reportedly due to neural, circulatory, and thermoregulatory responses [6,8]. WBV has been shown to produce analgesic effects for muscles, tendons, and fascia, in turn removing pain barriers during stretching exercises [6,8,13,14]. Further, acute WBV has also been shown to decrease muscle soreness following eccentric exercise [5,13] making it a valuable recovery tool as these can hinder recovery and decrease exercise performance.

Periods of excessive training and inadequate recovery can result in muscle soreness, fatigue, and even lead to overreaching or overtraining [15]. Overreaching results in short term decrements in performances with possible recovery in several days to weeks, while overtraining involves longer decrements in performance with recovery taking several weeks or months [15,16,17,18]. Monitoring symptoms of fatigue and soreness can help prevent athletes from overreaching while maintaining high training loads [18].

Not only do measures of physical properties of muscle tissue provide information in regard to an athlete’s recovery status, but also the implementation of subjective measures of recovery can be used to assess how the athlete is feeling. The Profile of Mood States (POMS) is a 65-item questionnaire with six categories of mood states (i.e., tension, depression, anger, vigor, fatigue, confusion), which is a validated tool for the detection of fatigue and overreaching in individual athletes [17,18,19] The Brief Assessment of Mood (BAM) is an abbreviated 6-item version of the POMS, which consists of single ratings of the six affective states [19]. The BAM correlates strongly with the POMS [20], requires less time for completion, and may be a more advantageous tool for eliciting immediate responses [21]. In relation to performance, mood state decrements typically precede any decreases in athletic performances [22,23]. Some of the more impactful mood states are tension, depression, and fatigue [23,24,25].

With WBV showing signs of decreasing soreness and fatigue it is possible that its use may enable athletes to train more effectively, in turn increasing their performances. To our knowledge, the effect of collegiate swimming athletes performing whole-body static stretching exercises in conjunction with WBV has not been investigated. The purpose of the current study was to compare athletes’ self-ratings of fatigue, BAM, and muscle soreness between conditions of static stretching performed with and without WBV.

## 2. Materials and Methods

### 2.1. Subjects

Nineteen NCAA-Division I swimmers participated in this study completing two familiarization and six experimental sessions over seven weeks. All subjects were medically cleared for intercollegiate athletic participation, had the risks and benefits explained to them beforehand, signed an institutionally approved consent form to participate, and completed a medical history form. The University’s Institutional Review Board for Human Subjects approved all procedures. Data from baseline demographics and physical characteristics of subjects are included in Table 1.

### 2.2. Procedures

To determine if differences existed between SS and WBV+SS conditions, a mixed factorial experimental design was employed. NCAA-Division I distance and sprint swimmers were recruited to participate. The SS and SS+WBV groups were counterbalanced by gender and swim event (distance or sprint). Session 1 included body composition testing (i.e., Bod Pod) and familiarization with the static stretching (SS) protocol of 9 different exercises (15 total exercises) as well as the WBV protocol. Session 2 included familiarization with the self-rating scales of BAM, fatigue, soreness, and ratings of perceived exertion (RPE) as well as practice of the aforementioned protocols. There were 6 total experimental sessions over a 6-week period. Following completion of experimental sessions 1–3, there was a 2-week washout period before subjects crossed over to the next condition (SS, WBV+SS) for experimental sessions 4–6. The six experimental sessions occurred immediately post-swim practice at the same time of day for each subject.

### 2.3. Body Composition

Subjects were instructed to drink only water and not to eat or exercise for the preceding two hours. Upon arrival to the laboratory, height and body mass were recorded to the nearest 0.01 cm and 0.02 kg, respectively using a stadiometer and digital scale (Bod Pod; Cosmed, Chicago, IL, USA) calibrated according to manufacturer guidelines with subjects bare foot. Body composition was then assessed using air displacement plethysmography (Bod Pod; Cosmed, Chicago, IL, USA) calibrated according to manufacturer guidelines. Lycra and swim caps were worn during testing. Jewelry was removed prior in accordance with standard operating procedures in order to reduce air displacement. A trained Bod Pod technician performed all testing. Previous studies indicate air displacement plethysmography to be an accurate and reliable means to assess changes in body composition [26]. Body mass and body volume were then used to estimate body fat percentage (%BF) based upon the Brozek equation for men and women [27].

### 2.4. Coach’s Ratings of Practice Difficulty

During practice the head swim coach was asked to rate the difficulty of practice on a 1–5 scale as follows: 1–1.5 = very easy; 2–2.5 = easy; 3–3.5 = moderate; 4–4.5 = hard; and 5 = very hard. Each practice was rated for the experimental period by the head swim coach who has approximately 20 years of head swim coaching experience.

### 2.5. Self-Rating Scales

In accordance with previously published methods [22], all subjects read instructions and practiced (i.e., familiarization session 2) using the self-rating scales of Overall Fatigue (FAT), Soreness (SOR), Borg CR-10 (RPE), and the Brief Assessment of Mood (BAM). Ratings for FAT and SOR are included in Table 2. The BAM is an abbreviated version [24] of the 65-item POMS [23]. The rating scale and six affective states of the BAM are represented in Table 3.

### 2.6. Whole-Body Vibration and Static Stretching Program

WBV was administered via a vibration platform (Pro5 AIRdaptive; Power Plate, Irvine, CA, USA) that produced vertical sinusoidal vibrations with a frequency range of 25–50 Hz and a vertical displacement range of 2–6 mm (i.e., amplitude). In familiarization session 1, subjects were introduced to the SS program (Table 4) by performing each exercise for 30 s on the WBV platform without vibration. In familiarization session 2, each subject practiced the SS protocol with vibration (frequency: 50 Hz, amplitude: 6 mm). The athletes had a high level of familiarity with the SS exercises because they performed them regularly as part of their sport training. However, none had previously used WBV. Total WBV exposure was 7.5 min per session during the WBV+SS condition.

### 2.7. Experimental Protocol

Following the two familiarization sessions, athletes returned to the laboratory on six separate occasions over six weeks to perform the experimental testing protocols (i.e., SS; WBV+SS). Experimental sessions 1–3 occurred over a 2-week period followed by a 2-week washout period during which all swim training continued. Upon completion of the washout period, subjects returned to the laboratory for a second 2-week period to perform the other condition during experimental sessions 4–6. All sessions were held in the morning (~10:00 a.m.) immediately following swim practice. Upon arrival to the laboratory, the subjects were provided a pencil and copies of the BAM, FAT, and SOR assessments. They sat apart from each other and were not permitted to talk with each other or leave their seats. Upon completion, subjects placed the assessments into an envelope. This procedure was also repeated immediately post condition. The same researchers administered the assessments during all experimental sessions. The BAM, FAT, and SOR were administered in pre and post conditions for each experimental session. The RPE was administered post condition. In between the pre and post self-ratings, subjects completed 7.5 min of SS exercises in the WBV+SS or SS condition. They worked in groups of two, held each SS exercise for 30 s, and alternated between the SS exercise and rest (1:1 work:rest ratio).

### 2.8. Statistical Analysis

All data were normally distributed as determined by the Kolomogrov-Smirnov test of normality. Self-ratings of FAT, RPE, SOR, and the 6 BAM affective states between conditions (SS and WBV+SS) were assessed by a mixed factorial (condition [two levels] × day [three levels] × pre/post [two levels]) analysis of variance. Least Squares Difference (LSD) post hoc analyses were performed when a significant finding (*p* ≤ 0.10) was identified. Bivariate (Pearson) correlations were computed to determine significant relationships among variables of interest. Alpha was set at *p* ≤ 0.10 for statistical significance. All analyses were conducted using the Statistical Package for the Social Sciences (SPSS, Version 21.0; SPSS Inc., Armonk, NY, USA).

## 3. Results

**Figure 1:**
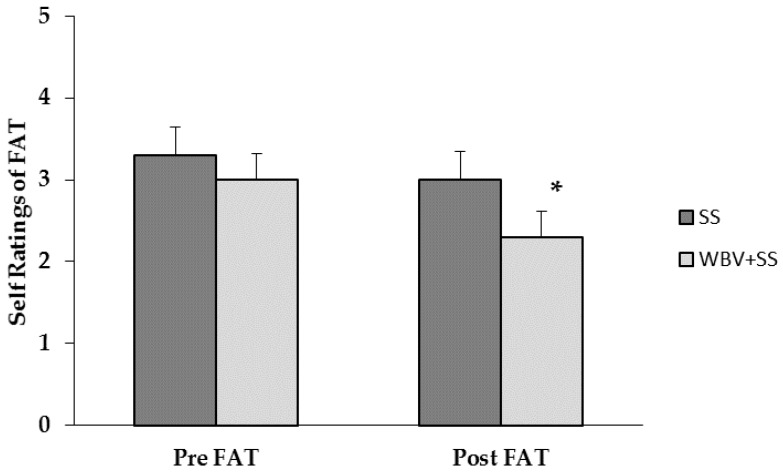
Pre and Post session FAT ratings by condition. Data are mean ± SD. * *p* = 0.002, WBV+SS versus SS. FAT: fatigue rating.

The head swim coach’s ratings of practice difficulty were as follows for the 11 swim practices during the experimental time period: phase I period, 4.0, 4.0, and 4.5 (*n* = 3, mean ± SD = 4.17 ± 0.29); washout period, 3.5, 4.0, 5.0, 4.5, 5.0 (*n* = 5, mean ± SD = 4.40 ± 0.65); and phase II period, 4.0, 4.5, 4.0 (*n* = 3, mean ± SD = 4.17 ± 0.29)). A significant condition by pre/post interaction was observed (*F*_2,18_ = 5.182, *p* = 0.035. Post hoc tests indicated the Post ratings of FAT were lower (*p* = 0.002) than Pre ratings for the WBV+SS condition (Figure 1). The BAM ratings were also significantly different between conditions (condition × pre/post, *F*_5,90_ = 2.312, *p* = 0.050). The WBV+SS elicited a reduction in tension (*p* = 0.031) and fatigue (*p* = 0.087) compared to SS (mean difference = 3.07 ± 0.131) (Table 5). There was a significant condition by pre/post interaction in SOR ratings (*F*_1,18_ = 5.341, *p* = 0.033). However, post-hoc analyses demonstrated no significant difference between conditions at either time point. A bivariate (Pearson) correlation elicited a strong positive relationship (*r* = 0.905, *p* = 0.013) between mean Post Tense (BAM) ratings and overall rating of practice difficulty.

## 4. Discussion

In order to identify differences between the WBV+SS and SS conditions, the self-ratings of FAT, SOR, and BAM (i.e., 6 affective states) were measured during six experimental sessions held post-swim practice over 6-week period during pre-season. The findings demonstrated a significant reduction in two out of six BAM measures as well as ratings of FAT. Typically, the monitoring of mood states or other psychological variables in conjunction with athletic performance has been focused upon sports in which athletes train with varying volumes leading up to a taper as the season ends [19,28]. In the current study, the athletes were finishing their pre-season training in preparation for the start of their competitive season, which resulted in increased training volumes. Therefore, it is possible that they may have begun to feel fatigued and tense due to the increased training loads. However, they likely were not far enough into the competitive season to show additional symptoms of overreaching.

Increasing the training load is commonly used as a means to enhance the athlete’s performance. Such intensification may lead to feelings of fatigue and possible decrements in performance, but if followed by adequate rest it often results in improved performance [15]. This process is known as functional overreaching and recovery periods are short and often planned, which is commonly referred to as de-loading [18,29]. Some coaches believe this temporary functional overreaching followed by planned recovery periods is necessary for improvements in performances [22]. However, due to the unpredictability of overreaching, others recommend that it should be avoided [30]. If the training load becomes overpowering and adequate recovery is not possible, non-functional overreaching or overtraining can occur [16,17], and complete recovery can take from months to years depending upon the severity of the condition. Therefore, consistent monitoring of athletes is advantageous for detecting signs of overreaching.

The BAM, a valid 6-item scale, was created in order to abbreviate the 65-item POMS instrument, and is superior to POMS for obtaining immediate psychological responses [21]. Psychological states are typically monitored in sports with changing training loads leading to a taper [28]. Mood state responses change in a linear fashion along as do training loads [19,22,23]. Therefore, increases in training loads will often be followed by an increase in mood disturbances [19,26,28]. In the present study overall practice difficulty, which was recorded by the head swim coach, fluctuated minimally on the days of the six experimental sessions with the overall mean of 4.17 the same for the experimental phase 1 and phase 2 periods. However, there was a small increase in coach’s ratings during the washout period (mean ± SD = 4.40 ± 0.65) before the athletes switched experimental conditions (WBV+SS, SS). This may have indicated a small overload in training, which is often observed in swimmers’ training programs in an attempt to elicit performance enhancements [25]. It has been reported previously that both swimmers and endurance athletes may find the overload in training to cause distress, thereby resulting in performance decrements [25].

As student-athletes, collegiate athletes have many daily demands placed upon their time in addition to consistent sport training. When the extraneous psychological stressors (e.g., extracurricular activities, work, school) are removed, mood state has been shown to be unaffected by large increases in training loads [31]. However, short term performance decrements may still occur without negative psychological symptoms [16]. In most cases, a decline in athletic performance will be preceded by a negative change in mood state [22,23]. Optimal performance and overreaching are separated by a fine line, especially when considering individual psychological factors [16]. Previous research with swimmers indicates tension, depression, and fatigue will increase with overload training [23,24,25]. The present study did not see any significant variation in pre-condition mood states, indicating that all athletes perceived practice difficulty in a similar fashion. However, the WBV+SS condition showed a significant reduction in the BAM items of *tense* and *fatigued* post-condition, which lends support to the premise that WBV+SS has a positive effect on mood state and may assist as a recovery aid or preventative measure against overreaching. The positive effect of WBV+SS is also supported by the correlation between post-condition *tense* ratings and practice difficulty. There was an increase in coach’s rating of training difficulty (i.e., 4.5) followed by a slight decrease in difficulty rating (i.e., 4.0) during the second round of experimental sessions that followed the washout period. O’Conner et al. previously reported *tense* ratings remained elevated in female swimmers after periods of overloading [25]; therefore, the use of WBV+SS to decrease feelings of tension following overloading is worthy of consideration.

Fatigue ratings are considered to be the most sensitive for early detection of overreaching in athletes because an athlete’s perception of fatigue has a tendency to be followed by performance decrements [17,18]. Soccer athletes who reported a higher level of fatigue experienced decreases in their maximal power output performances the following week [32]. Hooper et al. found POMS ratings (e.g., fatigue, confusion) to assist in predicting changes in race time performance in elite swimmers [33]. In the current study, the WBV+SS condition elicited lower ratings of fatigue on the FAT rating scale indicating that WBV+SS may be effective for attenuating post-practice fatigue.

WBV has elicited increases in flexibility, which could be due to acute analgesic effects [1,5,6]. The thermal effect of WBV plays a role in the acute improvements in flexibility, likely due to decreased muscular stiffness [5,6]. An increase in blood flow, from the thermal effect, may improve the removal of pain substrates and reduction of swelling. Previously published research has indicated regular WBV to have positive effects on muscle soreness, following eccentric exercise, when using a similar soreness scale to that of the present study [5,34,35]. In the current study, no differences were observed post-condition for ratings of muscle soreness on the SOR rating scale, which may be a result of the differing exercise selection and WBV protocols used in previous studies. The WBV protocol (7.5 min, frequency: 50 Hz, amplitude: 6 mm) in the current study was within what has been previously recommended to mimic deep tissue massages for pain reduction and recovery, [14,34]. Further research that addresses the effects of WBV+SS as a post-exercise recovery method is warranted. Taking the qualitative readings multiple times after sessions and using different exercises as well as WBV frequencies and durations are worthy of consideration.

The current study was conducted over a relatively short period (pre-season) with gradually increasing training volumes. In a sport like swimming where training loads often vary, leading to a taper at the end, it would be of interest to follow an entire season. Additionally, it is suggested that RPE ratings from individual athletes during training be examined. When comparing to individual mood states it may be of greater benefit to have athletes’ individual perceptions of practice difficulty in addition to an overall practice rating supplied by the head coach. It has been shown that mood state testing may be more conducive for detection of overreaching on an individual basis [17]. Further, calculating the volume of work in practice sessions may serve to elicit a clearer understanding of the difficulty level that sport training places on the athlete.

## 5. Conclusions

A post-exercise WBV+SS protocol, which consisted of nine, whole-body stretches, was sufficient to reduce self-ratings of fatigue in collegiate swimmers compared to SS without WBV. The condition of WBV+SS resulted in decreased feelings of *tense* and *fatigue* when assessed as part of mood state. This is of interest because mood state is a well-established indicator of how an athlete recovers from training. Incorporating a 15-min post-workout program of whole-body static stretching performed concurrently with WBV as part of athletes’ training may enhance the ability to recover from exercise.

## Figures and Tables

**Table 1:** Physical characteristics of subjects.

Characteristic	Men (*n* = 10)	Women (*n* = 9)
Age (year)	19.7 ± 1.0	19.3 ± 1.3
Height (cm)	183.0 ± 1.0 *	171.0 ± 5.7
Body Mass (kg)	77.1 ± 4.2 *	67.6 ± 7.2
%BF	13.1 ± 2.2 *	26.6 ± 4.1
Distance Swimmers (n)	6	5
Sprint Swimmers (n)	4	4

Data are mean ± SD. %BF: %body fat. *: *p* ≤ 0.05.

**Table 2:** Overall fatigue (FAT) and soreness (SOR) rating scales.

Rating	Description (FAT)	Rating	Description (SOR)
0	No Fatigue	0	No Soreness
1	Very Light Fatigue	1	Very Light Soreness
2	Moderate Fatigue	2	Moderate Soreness
3	Light (weak) Feeling of Fatigue	3	Light (weak) Feeling of Soreness
4		4	
5	Heavy (strong) Feeling of Fatigue	5	Heavy (strong) Feeling of Soreness
6		6	
7	Very Heavy Feeling of Fatigue	7	Very Heavy Feeling of Soreness
8		8	
9		9	
10	Maximal Fatigue	10	Maximal Soreness

**Table 3:** Brief assessment of mood (BAM).

Item	Not at All	A Little	Moderately	Quite a Bit	Extremely
Tense	0	1	2	3	4
Depressed	0	1	2	3	4
Angry	0	1	2	3	4
Vigorous	0	1	2	3	4
Fatigued	0	1	2	3	4
Confused	0	1	2	3	4

**Table 4:** Static stretching (SS) exercises performed with and without whole-body vibration (WBV).

SS Exercise Order	30 s Each
Hamstring	
Lower Back	
Quadriceps	R,L
Calf	R,L
Groin	
Hip	R,L
Pectoralis	R,L
Triceps	R,L
Shoulder	R,L

All stretches were held for 30 s. Where indicated both right (R) and left (L) limb were included.

**Table 5:** Mean mood state differences between static stretching with whole body vibration (WBV+SS) and static stretching with no whole body vibration (SS).

Mood States	Mean Difference	*p*-Value
Tense	0.307 ± 0.131	0.031 *
Depressed	0.193 ± 0.136	0.174
Angry	0.061 ± 0.108	0.578
Vigorous	0.105 ± 0.120	0.393
Fatigued	0.342 ± 0.189	0.087 *
Confusion	0.026 ± 0.023	0.268

* *p* ≤ 0.10 WBV+SS versus SS.
